# Acupuncture Reduced the Risk for Insomnia in Stroke Patients: A Propensity-Score Matched Cohort Study

**DOI:** 10.3389/fnagi.2021.698988

**Published:** 2021-08-13

**Authors:** Xuan Qiu, Nan Sheng Han, Jie Xiao Yao, Fang Rui Yu, Yan Yan Lin, Xun Zhuang

**Affiliations:** ^1^Acupuncture and Rehabilitation Clinical School, Guangzhou University of Traditional Chinese Medicine, Guangzhou, China; ^2^Department of Traditional Chinese Medicine, The Second Clinical Medical College, Guangdong Medical University, Dongguan, China; ^3^The First Affiliated Hospital of Chinese Medicine, Guangzhou University of Chinese Medicine, Guangzhou, China

**Keywords:** acupuncture, insomnia, stroke-rehabilitation, risk, cohort study

## Abstract

**Background:** Post-stroke insomnia (PSI) affects the quality of life for stroke patients, reduces the likelihood of successful rehabilitation, and produces additional complications following stroke. Previous reports have provided some information regarding PSI risk factors, but little is known concerning protective factors for PSI. This study analyzed the relationship between acupuncture and insomnia in stroke patients and explored the use of acupuncture as a preventive treatment.

**Methods:** Patients diagnosed with stroke from 2010 to 2019 were identified in the case database of the First Affiliated Hospital of Guangzhou University of Chinese These patients followed until 2020, and numerous factors were examined, including gender, age, stroke type, stroke location, and baseline comorbidities. A 1:1 propensity score was used to match an equal number of patients receiving acupuncture with stroke patients who did not receive acupuncture (*N* = 1,680 for each group). The purpose of the study was to compare the incidence of insomnia in these two stroke cohorts. We used the Cox regression model and Kaplan-Meier method to estimate the risk of insomnia as the outcome event.

**Results:** Compared with the non-acupuncture cohort in general, stroke patients who received acupuncture treatment exhibited a lower risk of insomnia after adjusting for age, gender, stroke type, stroke location, and comorbidities (adjusted hazard ratio *HR* = 0.27, 95% confidential interval = 0.23 to 0.32). Acupuncture also reduced the risk of PSI for both genders. The respective risks were *HR* = 0.28 (adjusted) for males and *HR* = 0.26 (adjusted) for females. Acupuncture also lowered the risk for PSI for different age groups. The risks were *HR* = 0.22 (adjusted) for individuals 18 to 39 years of age, *HR* = 0.31 (adjusted) for individuals 40 to 59 years of age, *HR* = 0.28 (adjusted) for those 60 to 79 years of age, and *HR* = 0.18 (adjusted) for individuals 80 years of age and older. Concerning the stroke type, regardless of whether the stroke was ischemic, hemorrhagic, or a combination of the two stroke types, patients who received acupuncture exhibited lower risk (adjusted *HR* = 0.28, 0.17, and 0.49, respectively). Concerning stroke location, except for the cerebral hemispheres (adjusted *HR* = 1.10, 95% confidential interval = 0.12 to 1.01), the risk of PSI after receiving acupuncture was lower for the frontal lobe (adjusted *HR* = 0.42), the basal ganglia (adjusted *HR* = 0.22), the radiation crown (adjusted *HR* = 0.42), the diencephalon (adjusted *HR* = 0.20), or multiple partial strokes (adjusted *HR* = 0.26), the risk of PSI after receiving acupuncture was lower. For all baseline complications, acupuncture reduced the risk of insomnia. The cumulative incidence of insomnia in the acupuncture cohort was significantly lower than the non-acupuncture cohort (log-rank test, *P* = 0.000).

**Limitations:** First, our research only included patients from a single center. Second, we did not classify the post-stroke insomnia severity. Second, the information was extracted manually. Overall, the sample size was small, and we needed to increase the sample size to strengthen the conclusions.

**Conclusion:** Acupuncture treatment reduced the risk of insomnia in stroke patients. Future research be conducted with increased sample sizes and further elaboration on the specific acupuncture protocols that were used.

## Introduction

Stroke patients typically experience at least one or more new or worsening symptoms of sleep disturbance, collectively referred to as post-stroke sleep disorder (PSSD) (Lisabeth et al., 2017). PSSD is a common complication after stroke, and is primarily divided into breathing-related obstructive sleep apnea syndrome or sleep disorders not related to breathing, such as periodic limb movement disorder, REM sleep behavior disorder, and insomnia (Pajediene et al., 2020). Post-stroke insomnia (PSI) is one of the most common types of sleep disorders. Approximately 37 to 59% of stroke experience insomnia (Duss et al., 2018). PSI is common in the six months before the occurrence of stroke, and when the PSI exceeds one year, the patient is considered to exhibit both objective and subjective insomnia (Suh et al., 2016). The objective and subjective sleep disorders occurring after stroke are detrimental to all stages of stroke, and especially for patients with mild to moderate stroke (Kim et al., 2020). Also, sleep parameters are closely related to the degree of physical function and quality of life for stroke patients (Kim et al., 2020). What is more intriguing is that stroke patients often have both PSI and post-stroke mental disorders such as anxiety and depression. Studies have reported that the possibility of anxiety, depression, and fatigue occurring in chronic recurrent insomnia groups was remarkably increased (Baylan et al., 2020). In addition, PSI impaired daily living activities during a one-month rehabilitation process (Glozier et al., 2017; Huang et al., 2018). These impairments primarily manifested in patients as a lower quality of life, decreased ability to carry out daily living activities, and affecting energy and cognitive ability (Tang et al., 2015; Lisabeth et al., 2019). Severe insomnia can double the risk of stroke; the results of a prospective cohort study with a follow-up as long as six years indicated that PSI was a risk factor for stroke recurrence and might be directly related to stroke mortality (Li et al., 2018). In the sixth year of the study, the risk of death had increased by 1.66 times (Li et al., 2018). Unfortunately, the incidence of PSI has been underestimated clinically because the assessment of sleep disorders after stroke usually is performed a considerable time after the stroke occurred (Wessendorf et al., 2000). In recent years, researchers have discussed the relationship between insomnia and stroke and realized that severe sleep disorders were related to poor long-term functional prognoses after stroke (Wessendorf et al., 2000). However, treatment for insomnia has remained limited. Drug intervention has continued to be a standard treatment method for PSI. However, in view of drug efficiency and safety, long-term follow-up and evaluation of drug treatments for PSI are needed (Terzano et al., 2003). In addition, some studies have pointed out that drug treatment for insomnia might increase the risk of dementia and stroke (Chen et al., 2012; Bassetti et al., 2020). Therefore, it is urgent to find additional effective and feasible treatment methods for PSI.

Acupuncture is a treatment method that cures disease by stimulating acupoints on the meridians. The meridians form an extensive network system, which is a pathway through which the human body runs Qi and blood, communicates with the internal organs, communicates internally and externally, and how energy flows throughout the body. Meridians crisscross and spread over the body, circulate the blood and nourish the body. Acupuncture points are specific locations for the infusion of Qi from the viscera, meridians, and collaterals of the body. They are the reaction points for diseases and also the stimulation points for acupuncture treatment of disease. The acupoints are not isolated from acupuncture points; they are closely related to the deep tissues and organs and are three-dimensional structures for circulating blood. Therefore, when the human body is diseased, the corresponding meridians and acupuncture points have a specific response on the body surface. Stimulation of these specific locations using acupuncture can cure the disease in the body.

In 2004, Kim et al. compared the use of sham and actual acupuncture and found that the actual acupuncture treatment for PSI achieved significantly better improvement (Kim et al., 2004). In 2009, Lee and other researchers used more objective detection methods (24-h ambulatory electrocardiograms and ambulatory blood pressure measurements) to affirm the efficacy of acupuncture in PSI treatment and discussed its specific mechanism (Lee et al., 2009). Acupuncture primarily affects the autonomic nervous system to restore its function to a balanced state (Kotani et al., 2001). This action is primarily manifested as inhibition of the excitability of the sympathetic nerves and stabilizing their hyperactivity (Kotani et al., 2001). This also is what Chinese medicine calls the “balance of yin and yang, life activities being normally maintained.”

## Methods

### Data Source

This was a clinical retrospective study based on patient information obtained from the First Affiliated Hospital of Guangzhou University of Chinese Medicine. The study was approved by the hospital's institutional review board. Our research mainly observed patients who were definitively diagnosed with stroke at the First Affiliated Hospital of Guangzhou University of Chinese Medicine from 2010 to 2020. The patient's clinical data were obtained from the electronic medical records system. The diagnostic codes that were used were contained in the International Classification of Diseases, Tenth Revision, Clinical Modification (ICD-10-CM).

The patient selection criteria included: (1) 18 years of age or older; (2) the stroke met the diagnostic criteria provided by the World Health Organization, was supported by explicit imaging evidence (cranial CT or MRI), and the clinical symptoms were present more than 24 h; (3) the stroke was diagnosed before insomnia; (4) acupuncture treatment in the acupuncture cohort occurred more than one time.

### Study Subjects

**Figure 1:**
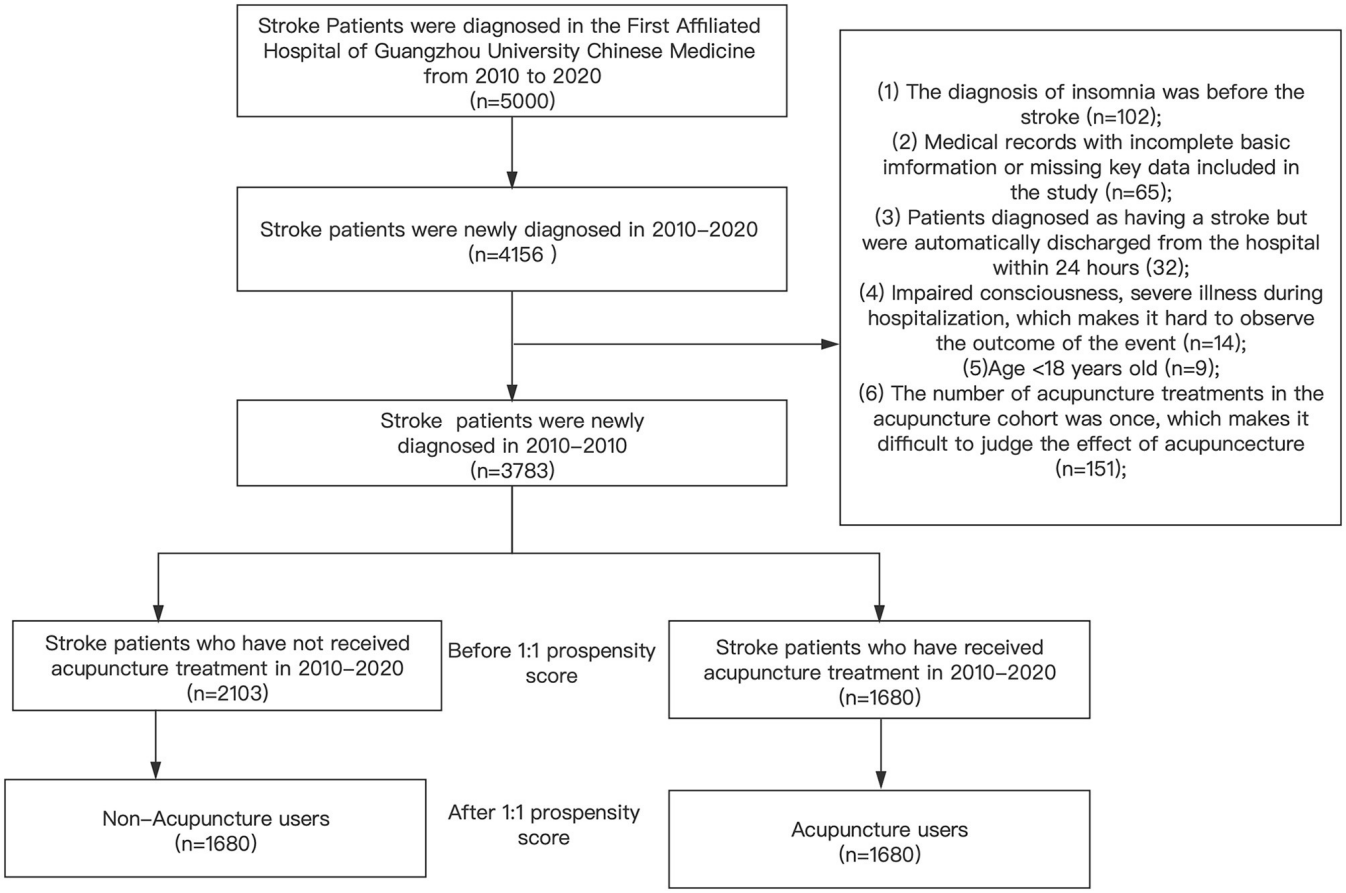
This figure shows the theoretical basis of the entire research and the specific operation process.After matching the two cohorts with the 1:1 propensity score matching method, in the end, there were 1680 patients in the acupuncture cohort and the non-acupuncture cohort.

We included all stroke patients classified as having ICD-10-CM: I61, I62, I63, I64, I65, and I66 at the First Affiliated Hospital of Guangzhou University of Chinese Medicine from January 1, 2010 to December 31, 2020 (*N* = 5,000). A total of 4,156 patients were newly diagnosed with stroke. The exclusion criteria included the following: (1) The diagnosis of insomnia ICD-10-CM:F51 before stroke, including I61, I62, I63, I64, I65, and I66; (2) the medical record did not contain all the necessary basic information or essential data were missing in the included study; (3) patients were diagnosed as having had a stroke, but were automatically discharged from the hospital within 24 h and whose outcome was not observed; (4) patients experienced severe illness during hospitalization that made it impossible to observe the outcome events; (5) the patient's age was less than 18 years; (6) only one acupuncture treatment occurred for the patient in the acupuncture cohort and the effect of acupuncture could not be ascertained; (7) The stroke patients with a previous history of insomnia. Under the guidance of the exclusion criteria, a total of 1,680 stroke patients (*N* = 3,783) who received acupuncture treatment after the first diagnosis of stroke were defined as the acupuncture cohort. To ensure the two cohorts were comparable, we matched the two cohorts according to the patient age, gender, stroke type, stroke location, whether they received acupuncture, and all baseline comorbidities by using the 1:1 propensity score matching method, the matching value was set to 0.01, and 3,360 patients were matched. Thus, 1,680 patients received acupuncture, and 1,680 patients did not (Figure 1).

### Covariate Assessments

In our study, the following covariate factors were included in the analysis

(1) Basic patient information, including name, medical record ID, gender, age, and date of admission;
(2) Information related to stroke, including time of first stroke, stroke type (ischemic stroke or hemorrhagic stroke or mixed).stroke location (frontal lobe, basal ganglia, corona radiation, cerebral hemisphere, diencephalon, or multiple sites).
(3) Information related to the acupuncture procedure that included the acupuncture exposure factor for which observation was divided into the acupuncture cohort group, non-acupuncture cohort group, and the follow-up that was recorded in the acupuncture cohort.
(4) Baseline diseases, including any previous hypertension (ICD-10-CM: I10), diabetes (ICD-10-CM: E10-14), hyperlipidemia (ICD-10-CM: E78), mental disorders (ICD-10-CM: F31, F32, F34, and F41), repeated strokes, coronary atherosclerotic cardiomyopathy (ICD-10-CM: I25.1), chronic atrophic gastritis (ICD-10-CM: K29.3, K29.4), constipation (ICD-10-CM: K59.0), and carotid atherosclerosis (ICD-10-CM: I70.8) were recorded.

### Outcome Measurements

We considered insomnia to be an end-point event in the two stroke cohorts after the end of the follow-up time. The date for the acupuncture treatment after the first diagnosis of stroke was defined as the index day. The main goal of the study was to compare the risk of insomnia with cohort that received acupuncture and the cohort that did not receive acupuncture. A determination that insomnia was present primarily depended on whether the following drugs were listed in the patient's medical record information: benzodiazepine sedatives (midazolam, estazolam, alprazolam, clonazepam, or diazepam) and non-benzodiazepines (Zolpidem, or Zaleplon).

### Statistical Analysis

Categorical variables were analyzed using the chi-square test, Continuous variables using the Student's *t*-test to evaluate the difference in baseline characteristics between acupuncture cohort and non-acupuncture cohort. The Cox proportional hazard regression was used to evaluate the hazard ratios (HRs) with 95% confidence intervals (CIs). The Kaplan-Meier method and log-rank test were used to compare the difference in the cumulative incidence of insomnia during the follow-up period between the two cohorts. A *p*-value < 0.05 indicated statistical significance. All statistical analyses were performed using IBM SPSS software version 23.0.

## Results

As seen in Table 1, there were more males than females in both groups. The average age was 65 years, with the 60–79 age group as the largest group being patients aged 60 to 79 years. Concerning stroke type, ischemic stroke was the most common type of stroke, which accounted for 86.8% of the acupuncture cohort and 84.2% of the non-acupuncture cohort. The majority of strokes occurred at the site of the radiation crown, which was 18.5% in the acupuncture cohort and 18.6% in the non-acupuncture cohort. In the acupuncture group cohort, the average number of follow-up visits was 4.98. Also, gender, age, stroke type, stroke location, and baseline disease between the two groups revealed no statistical differences after matching (*P* > 0.05)

**Table 1:** Characteristics of stroke patients based on the factors of whether accepted acupuncture.

**Variable**	**Acupuncture treatment**				*p*-value^*^
	**No (n=1680)**		**Yes (n=1680)**		
	**n**	**%**	**n**	**%**	
**Gender**					0.203
Women	676	40.2	640	38.1	
Men	1,004	59.8	1,040	61.9	
**Age group**					0.075
18-39	45	2.7	38	2.3	
40-59	507	30.2	480	28.6	
60-79	864	51.4	935	55.7	
≥80	264	15.7	227	13.5	
Mean ± SD(years)^†^	65.43		65.56		
	13.21		12.65		
**Stroke type**					0.093
Ischemic stroke	1,458	86.8	1,415	84.2	
Hemorrhagic stroke	161	9.6	186	11.1	
Mixed types of stroke	61	3.6	79	4.7	
**Location of the stroke**					0.735
Frontal lobe	101	6.0	108	6.4	
Basal ganglia	310	18.5	313	18.6	
Corona radiation	310	18.5	313	18.6	
Cerebral hemisphere	63	3.8	74	4.4	
Diencephalon	75	4.5	61	3.6	
Multiple lesions	65	3.9	60	3.6	
**Baseline disease**				
Hypertension	1,054	62.7	1,072	63.8	0.519
Diabetes	416	24.8	421	25.1	0.842
Hyperlipidemia	645	38.4	607	36.1	0.175
Mental disorder	61	3.6	43	2.6	0.073
Repeated strokes	447	26.6	410	24.4	0.143
Coronary atherosclerotic heart disease	406	24.2	361	21.5	0.064
Constipation	755	44.9	742	44.2	0.652
Chronic atrophic gastritis	137	8.2	112	6.7	0.100
Carotid atherosclerosis	745	44.3	698	41.5	0.101
Duration between insomnia date and index, days (mean, median)	98 (15)		230 (41)		
Time from first acupuncture to stroke days (mean, median)	-		30.7 (7)		
Acupuncture visits (mean, median)	-		4.98 (2)		

*The average (median) follow-up period of insomnia in the acupuncture cohort and non-acupuncture cohort were 498 (15) days and 230 (41) days, respectively*.

*In the acupuncture cohort, the average (median) was 30.7 (7) days respectively for index days*.

*In the acupuncture queue, The mean (median) of the follow-up period were 4.98 (2)*.

* *Chi-squared test*;

† *t-test*.

*Repeated strokes are defined as more than one stroke*.

*Mental disorder includes anxiety and depression*.

*Mixed type of stroke includes ischemic type and Hemorrhagic stroke*.

Insomnia was observed in 721 stroke patients. We used the crude HR (the risk value of a single variable) and the adjusted HR (the risk value of the variable after adjustment) to evaluate the influence of a range of factors on PSI (the outcome event). As seen in Table 2, both before and after the adjustment, when compared with patients in the non-acupuncture cohort, patients in the acupuncture cohort exhibited a significantly lower risk of PSI (crude HR = 0.22, *P* = 0.000, and adjusted HR = 0.27, *P* = 0.000).

**Table 2:** Cox model with hazard ratios and 95% confidence intervals of each covariate that developed into insomnia after stroke.

**Variable**	**No. of event**	Crude*			**Adjusted^†^**		
	**(n = 721)**	**HR**	**(95% CI)**	***p*-value**	**HR**	**(95% CI)**	***p*-value**
**Acupuncture**							
No	500	1.00	reference		1.00	reference	
Yes	221	0.22	(0.19–0.26)	0.000	0.27	(0.23–0.32)	0.000
**Sex**							
Women	301	1.08	(0.93–1.26)	0.289	1.19	(1.02–1.39)	0.025
Men	420	1.00	reference		1.00	reference	
**Age group**							
18–39	22	1.54	(0.98–2.42)	0.064	2.02	(1.27–3.22)	0.003
40–59	191	1.09	(0.87–1.37)	0.441	1.36	(1.07–1.72)	0.012
60–79	382	0.91	(0.75–1.12)	0.383	1.06	(0.86–1.30)	0.611
≥80	126	1.00	reference		1.00	reference	
**Stroke type**							
Ischemic stroke	587	0.83	(0.60–1.13)	0.238	0.76	(0.55–1.05)	0.093
Hemorrhagic stroke	93	1.13	(0.78–1.63)	0.525	1.24	(0.85–1.81)	0.269
Both types of stroke	41	1.00	reference		1.00	reference	
**Location of the stroke**							
Frontal lobe	48	1.22	(0.91–1.65)	0.188	1.26	(0.93–1.71)	0.137
Basal ganglia	134	1.25	(1.03–1.51)	0.026	1.11	(0.91–1.35)	0.316
Radiation crown	31	1.27	(1.03–1.51)	0.201	1.07	(0.74–1.55)	0.721
Cerebral hemisphere	35	1.24	(0.88–1.83)	0.226	0.90	(0.63–1.29)	0.579
Diencephalon	28	1.15	(0.79–1.69)	0.473	0.83	(0.55–1.23)	0.346
Multiple lesions	445	1.00	reference		1.00	reference	
**Baseline disease**							
Hypertension	539	1.71	(1.44–2.02)	0.000	1.70	(1.43–2.02)	0.000
Diabetes	284	1.59	(1.37–1.85)	0.000	1.45	(1.24–1.69)	0.000
Hyperlipidemia	410	2.07	(1.78–2.40)	0.000	1.41	(1.21–1.66)	0.000
Mental disorder	71	3.50	(2.74-4.47)	0.000	3.34	(2.60–4.31)	0.000
Repeated strokes	431	2.37	(2.02–2.76)	0.000	1.50	(1.26–1.78)	0.000
Coronary atherosclerotic heart disease	204	1.13	(0.96–1.33)	0.146	0.94	(0.79–1.11)	0.456
Constipation	407	1.41	(1.22–1.63)	0.000	1.02	(0.87–1.18)	0.848
Chronic atrophic gastritis	79	1.36	(1.08–1.72)	0.009	1.04	(0.82–1.32)	0.76
Carotid atherosclerosis	521	3.32	(2.82–3.91)	0.000	2.50	(2.10–2.99)	0.000

*Adjusted HR: adjusted hazard ratio: mutually adjusted for Age, gender, stroke type, stroke site, and baseline diseases: hypertension, diabetes, hyperlipidemia, psychological disorders, repeated strokes, coronary heart disease, constipation, chronic atrophic gastritis, cervical sclerosis in Cox proportional hazards regression*.

*Crude HR: relative hazard ratios*.

Before adjustment, there was no significant difference in risk between men and women (crude HR = 1.08, *P* = 0.289), but after adjusting for other covariates, women showed a higher risk than men (adjusted HR = 1.19, *P* = 0.025). Among the different age groups, when patients over 80 years of age were compared to the other groups, the 18 to 39- and 40 to 59-year-old groups exhibited higher risk after adjustment (adjusted HR = 2.02, *P* = 0.003), (adjusted HR = 1.36 and *P* = 0.012, respectively). In contrast, when compared with the 60 to 79-year-old group, there was no discernible difference (adjusted HR = 1.06, *P* = 0.61). There were no differences between ischemic type, hemorrhagic type, or mixed types of stroke before or after adjustment (*P* > 0.05). Concerning the stroke location, compared with the multiple locations (imaging evidence demonstrated more than two sites of stroke sites), there were no significant differences in the frontal lobe, corona radiation, cerebral hemispheres, or diencephalon (*P* > 0.05). Only the basal ganglia showed a higher risk before adjustment (crude HR = 1.25, *P* = 0.026). However, after adjusting for the influence of other variables, no difference was observed (adjusted HR = 1.11, *P* = 0.316). Among the baseline comorbidities, hypertension (adjusted HR = 1.70, *P* = 0.000), diabetes (adjusted HR = 1.45, *P* = 0.000), hyperlipidemia (adjusted HR = 1.41, *P* = 0.000), mental illness (adjusted HR = 1.41, *P* = 0.000 for depression and HR = 3.34, *P* = 0.000 for anxiety), repeated strokes (adjusted HR = 1.50, *P* = 0.000), and carotid atherosclerosis (adjusted HR = 2.50, *P* = 0.000), all increased the risk for insomnia to varying degrees. However, constipation (crude HR = 1.41, *P* = 0.000) and chronic atrophic gastritis (crude HR = 1.36, *P* = 0.009) showed a higher risk before adjustment, but no significant differences were observed after adjustment. Constipation exhibited an adjusted HR = 1.02 (*P* = 0.848), and chronic atrophic gastritis exhibited an adjusted HR = 1.04 (*P* = 0.76). Concerning atrial fibrillation, no differences were observed for the crude HR = 1.13 (*P* = 0.146) or the adjusted HR = 0.94 (*P* = 0.456). All results are shown in Table 2.

**Figure 2:**
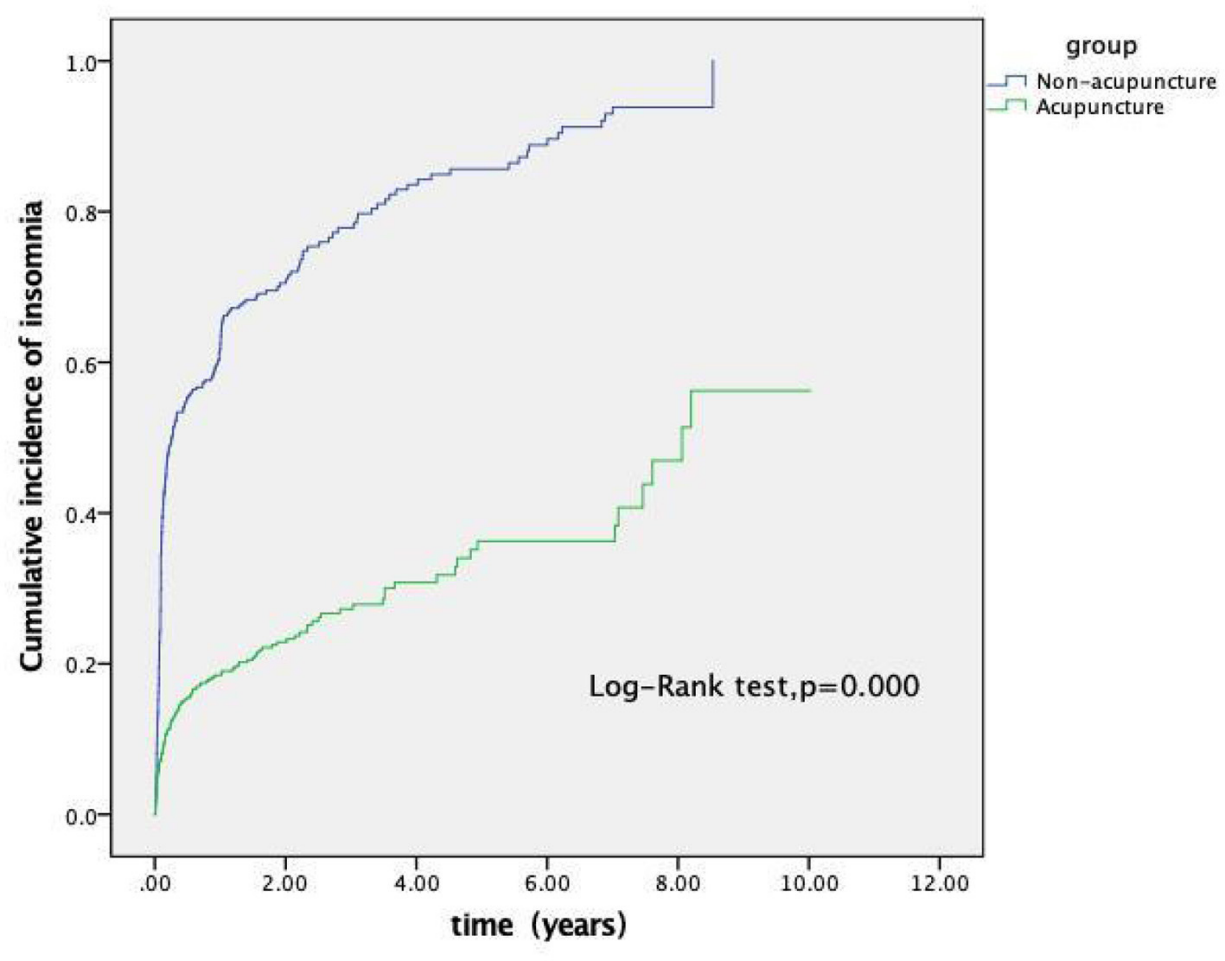
Cumulative incidence of insomnia between the acupuncture and non-acupuncture cohorts of stroke patients. As shown in the figure, the cumulative incidence of the acupuncture cohort was significantly lower than that of the non-acupuncture cohort (log-rank test, *p* = 0.000).

Compared with insomnia patients in the non-acupuncture group, regardless of gender, age group, stroke type, stroke site, and baseline comorbidities, the incidence and risk ratios for patients receiving acupuncture treatment were significantly lower. (adjusted HR <1). We also determined the overall risk associated with acupuncture and non-acupuncture in patients with insomnia. Table 3 shows that for patients of different genders, age groups, stroke type, stroke location, or baseline disease, the groups that received acupuncture showed a lower overall risk before and after adjusting for the effects of other variables. In addition, most of the patients showed a lower incidence, and the cumulative incidence in the acupuncture cohort was significantly lower than the non-user acupuncture group (log-rank test, *P* = 0.000, Figure 2).

**Table 3:** The respective hazard ratios and Incidence rates of the acupuncture and non-acupuncture cohorts before and after adjustment for gender, age, stroke type, stroke site, and baseline complications.

**Variables**	**Acupuncture treatment**						**Compared with non-acupuncture users**			
	**No (n=1680)**			**Yes (n=1680)**						
	**Event**	**Person**	**IR^†^**	**Event**	**Person**	**IR^†^**	**Crude HR**	***p***	**Adjusted HR**	***p***
		**Years**			**Years**		**(95% CI)**	**value**	**(95% CI)**	**value**
Total	500	2,24,397	0.22	221	1,512	0.15	0.22 (0.19–0.26)	0.000	0.27 (0.23–0.32)	0.000
**Sex**										
Men	284	1,39,808	2.03	136	99,013	1.37	0.24 (0.20–0.30)	0.000	0.28 (0.23–0.35)	0.000
Women	216	84,589	2.55	85	52,268	1.62	0.20 (0.15–0.25)	0.000	0.26 (0.20–0.34)	0.000
**Age group**										
18–39	16	1,706	9.38	6	369	16.26	0.32 (0.12–0.82)	0.017	0.22 (0.06–0.81)	0.013
40–59	129	34,797	3.71	62	22,747	2.73	0.23 (0.17–0.31)	0.000	0.31 (0.23–0.44)	0.000
60–79	260	1,30,961	1.99	122	89,649	1.36	0.24 (0.19–0.29)	0.000	0.28 (0.23–0.36)	0.000
≥80	95	56,931	1.67	31	38,515	0.80	0.15 (0.10–0.23)	0.000	0.18 (0.23–0.36)	0.000
**Stroke type**										
Ischemic stroke	409	1.94	0.19	178	1,36,120	1.31	0.23 (0.19–0.27)	0.000	0.28 (0.24–0.34)	0.000
Hemorrhagic stroke	66	8,723	7.57	27	9,367	2.88	0.15 (0.10–0.24)	0.000	0.17 (0.10–0.28)	0.000
Both types of stroke	25	5,098	4.90	16	5,794	2.76	0.30 (0.16–0.58)	0.000	0.35 (0.17–0.69)	0.002
**Location of the stroke**										
Frontal lobe	28	6,293	4.45	20	11,435	1.75	0.35 (0.19–0.63)	0.000	0.42 (0.22–0.82)	0.015
Basal ganglia	89	35,331	2.52	45	38,547	1.17	0.23 (0.16–0.34)	0.000	0.22 (0.15–0.33)	0.000
Corona radiation	23	12,427	1.85	8	10,016	0.80	0.18 (0.08–0.42)	0.000	0.35 (0.12–1.01)	0.071
Cerebral hemisphere	27	16,405	1.65	8	657	12.18	0.19 (0.09–0.43)	0.000	1.10 (0.12–1.01)	0.905
Diencephalon	19	6,060	3.14	9	4,682	1.92	0.23 (0.09–0.43)	0.001	0.20 (0.07–0.60)	0.004
Multiple lesions	314	1,47,879	2.12	131	85,942	1.52	0.21 (0.17–0.26)	0.000	0.26 (0.21–0.33)	0.000
**Baseline disease**										
**Hypertension**										
No	141	85,375	1.65	41	34,958	1.17	0.14 (0.10–0.19)	0.000	0.21 (0.15–0.31)	0.000
Yes	359	1,39,021	2.59	180	1,16,323	1.55	0.26 (0.21–0.31)	0.000	0.30 (0.25–0.36)	0.000
**Diabetes**										
No	304	1,23,265	2.47	133	81,295	1.64	0.21 (0.17–0.26)	0.000	0.29 (0.24–0.36)	0.000
Yes	196	1,01,131	1.94	88	69,986	1.26	0.24 (0.19–0.31)	0.000	0.27 (0.20–0.35)	0.000
**Hyperlipidemia**										
No	215	59,726	3.60	96	53,210	1.80	0.19 (0.14–0.23)	0.000	0.21 (0.16–0.27)	0.000
Yes	285	16,4671	1.73	125	98,071	1.27	0.28 (0.22–0.34)	0.000	0.41 (0.33–0.52)	0.000
**Mental disorder**										
No	451	2,17,969	2.07	199	1,32,972	1.51	0.22 (0.18–0.26)	0.000	0.29 (0.24–0.34)	0.000
Yes	49	6,427	7.62	22	18,309	1.20	0.14 (0.14–0.42)	0.000	0.29 (0.15–0.55)	0.000
**Repeated strokes**										
No	192	41,000	4.68	98	26,487	3.70	0.21 (0.16–0.27)	0.000	0.22 (0.17–0.29)	0.000
Yes	308	1,83,109	1.68	123	1,24,794	0.99	0.27 (0.22–0.33)	0.000	0.38 (0.31–0.47)	0.000
**Coronary atherosclerotic heart disease**										
No	354	1,29,098	2.74	163	1,04,000	1.57	0.22 (0.18–0.26)	0.000	0.26 (0.22–0.32)	0.000
Yes	146	95,298	1.53	58	47,246	1.23	0.24 (0.17–0.32)	0.000	0.31 (0.22–0.42)	0.000
**Constipation**										
No	226	89,038	2.54	88	48,652	1.81	0.17 (0.13–0.21)	0.000	0.19 (0.15–0.25)	0.000
Yes	274	1,35,358	2.02	133	1,02,630	1.30	0.29 (0.23–0.35)	0.000	0.36 (0.29–0.45)	0.000
**Chronic atrophic gastritis**										
No	446	1,86,805	2.39	196	1,26,271	1.56	0.22 (0.18–0.26)	0.000	0.27 (0.23–0.32)	0.000
Yes	54	37,591	1.44	25	25,010	0.10	0.29 (0.18–0.47)	0.000	0.34 (0.20–0.58)	0.000
**Carotid atherosclerosis**										
No	140	27,126	5.16	60	36298	1.65	0.18 (0.13–0.24)	0.000	0.17 (0.13–0.24)	0.000
Yes	360	1,97,271	1.82	161	1,14,983	1.40	0.28 (0.23–0.34)	0.000	0.35 (0.30–0.43)	0.000

*Abbreviations: IR, incidence rates, per 1000 person-years; HR, hazard ratio; CI, confidence interval*.

*Adjusted HR: adjusted for age, gender, diabetes, sex, age, stroke type, stroke location and baseline disease Hypertension, diabetes, hyperlipidemia, psychological disorders, repeated strokes, coronary heart disease, constipation, chronic atrophic gastritis, carotid atherosclerosis in Cox proportional hazards regression*.

## Discussion

Insomnia after a stroke may be directly caused by the pathological changes due to the stroke itself or indirectly caused by the resulting emotional disorders, disability, and changes in the external environment that result from the stroke. Regardless of the reason, identifying and resolving sleep disorders should be included in stroke prevention. If insomnia after a stroke failed to receive adequate attention and timely treatment, sleep disorders could increase the possibility of stroke patients developing repeated strokes, and severe sleep disorders could lead to suicidal ideation (Tang et al., 2011). At the same time, stroke increases the medical burden, nursing costs, and leads to decreases productivity (Mims and Kirsch, 2016). Therefore, the impact of insomnia on economic losses should not be underestimated. These findings should make clinicians aware of the potential harm that can result from insomnia. Several researchers have reported that acupuncture can effectively reduce PSI, but this information has been presented in the form of protocols that lacked research results (Xiang et al., 2020). Interestingly, past retrospective studies also have found that acupuncture exhibited advantages in treating diseases that developed as sequelae to stroke (Cao et al., 2016; Liao et al., 2020). Although the research topics were not precisely the same, our conclusions were consistent with these studies in that acupuncture was advantageous in treating stroke-related diseases.

This was a retrospective clinical study whose primary purpose was to explore the protective factors of PSI, because most previous studies focused on analyzing the risk factors related to stroke. For example, common risk factors for stroke include high blood pressure, diabetes, dyslipidemia, smoking, and alcohol consumption. It is rarely recognized that insomnia is closely related to the occurrence of stroke. In this context, the treatment options for PSI also are limited. Thus, changing the management of PSI and identifying protective factors also can be important means of preventing stroke. Our research focused on the relationship between acupuncture and insomnia in stroke patients. The results demonstrated that for different genders, ages, stroke type, stroke site, and baseline comorbidities, acupuncture treatment could significantly reduce the risk of insomnia in stroke patients. Thus, we have increased our understanding of stroke and emphasized disease prevention, which aligns with the scope of TCM disease prevention. We also increased our understanding of covariates that were related to stroke and explored the potential impact of stroke type, such us stroke location, coronary heart disease, carotid atherosclerosis, chronic atrophic gastritis, hyperlipidemia and other factors on PSI. These factors might be closely related to the occurrence of stroke or might be related to the outcome event. We have classified, analyzed, and summarized these factors and assessed their effects on insomnia after stroke. However, additional research is needed concerning these factors.

In our study, after adjusting for the influence of confounding covariates, it was noted that women exhibited higher risk for PSI (adjusted HR = 1.19, *P* = 0.025), which was similar to results reported by previous studies (Weng et al., 2016). This result might be related to endocrine changes in women and the relatively weaker ability of women to regulate stress, but the underlying mechanism is still unclear (Leppävuori et al., 2002). Overall, there were no consistent results regarding the influence of gender. One study indicated no difference in the risk of PSI between men and women (Chen et al., 2008). Another study also suggested that gender factors had no significant impact on PSI (Kim et al., 2020). Thus, larger sample sizes and multi-center clinical studies are needed to derive more definitive conclusions in the future. When different age groups were considered, compared with people over 80 years of age, the 18 to 39- (adjusted HR = 2.02, *P* = 0.003) and 40 to 59-year-old groups (adjusted HR = 1.36, *P* = 0.012) exhibited higher a risk for PSI after adjustment. This result was comparable with the clinical phenomena observed for stroke. One study of 18 to 45-year-old stroke patients investigated the risk factors for stroke onset (Sterr et al., 2008). The results from that study suggested that low-quality sleep was the fifth largest predictor of stroke in young people and was closely linked to the pathogenesis of stroke (Sterr et al., 2008). PSI primarily occurred in younger age groups, which may be related to the body's regulatory abilities for individuals in these age groups. Physiological and pathological changes after stroke were more intense at these ages, and insomnia was one of the manifestations of body regulation that was noted. These observations have provided suggestions for clinical treatment. Thus, young patients need to pay attention to their sleep quality, as it can be beneficial for stroke recovery. There was no difference in the risk of ischemic, hemorrhagic, and mixed types of strokes (*P* > 0.05). Notably, the impact of stroke types on PSI consistency has been a topic of interest to researchers. Most researchers have indicated that cerebral ischemia was a common type of stroke associated with PSI (Zhang et al., 2014). Some studies have shown that the incidence of insomnia in patients with ischemic stroke was more than 60% (Johnson and Johnson, 2010). However, other studies have reported that no clear correlation was observed between the location of the lesion in stroke patients and sleep disorders, and the sleep disorder was related to the other risk factors that have been identified for sleep disorders.

It has been reported that cerebral ischemia was caused by affecting sleep disorders in patients with other existing risk factors and not due to the destruction of specific central nervous system structures (Fisse et al., 2017). This observation also was consistent with our research results. The relationship between the type of stroke and PSI should be studied further so that improved screening could help identify people at risk and provide more targeted treatment.

Concerning stroke location, compared with multiple sites (imaging evidence of more than two stroke locations), there was no significant difference in the frontal lobe, radiation crown, cerebral hemispheres, and diencephalon (*P* > 0.05). Only the basal ganglia showed a higher risk before adjustment (crude HR = 1.25, *P* = 0.026), but after adjusting for the influence of other variables, there was no difference (adjusted HR = 1.11, *P* = 0.316). However, stroke that occurred in the basal ganglia was closely related to sleep disorders. Studies have reported that the basal ganglia regulate emotion-related functions through associations with the frontal lobe and thalamus (Arsalidou et al., 2013). Therefore, patients with strokes in the basal ganglia also could experience mental illness and symptoms of insomnia. This view supports our research results.

Among the baseline comorbidities, hypertension, diabetes, and mental illness are well-known risk factors. Not surprisingly, similar results also were seen in our research results for hypertension (adjusted HR = 1.70, *P* = 0.000), diabetes (adjusted HR = 1.45, *P* = 0.000), and mental illness (adjusted HR = 3.34, *P* = 0.000). It is worth noting that mental illness was more noticeable among these risk factors. In fact, PSI and post-stroke psychological disorders are closely associated with each other. Mental disorders are extremely common after stroke (Kim et al., 2020). Stroke can cause early sleep difficulties and induce persistent sleep disorders in chronic conditions (Kim et al., 2017). Insomnia and mental disorders often interact, and patients with insomnia tend to be more anxious or depressed. The occurrence of depression usually reduces the duration and quality of sleep. Some patients with mental disorders also have insomnia after taking psychotropic drugs. In summary, the effects of insomnia and mental disorders after stroke usually interact in a vicious cycle. In view of the extensive and far-reaching relationship between psychological disorders and PSI, follow-up research should study the specific pathogenesis in detail.

The results from this study demonstrated that repeated strokes (adjusted HR = 1.50, *P* = 0.000), carotid atherosclerosis (adjusted HR = 2.50, *P* = 0.000), and hyperlipidemia (adjusted HR = 1.41, *P* = 0.000) also were risk factors for PSI. We believe that our research highlighted these results. Past studies have found that hyperlipidemia and carotid atherosclerosis were risk factors for stroke (Zhang et al., 2014), but the impact on PSI has not been investigated. Constipation (crude HR = 1.41, *P* = 0.000) and chronic atrophic gastritis (crude HR = 1.36, *P* = 0.009) showed higher risk before adjustment. We explored these two specific factors in our research for several reasons. From the perspective of traditional Chinese medicine, there is a proverb, “If the stomach is not good, it is very likely that you will not sleep well.” Chinese medicine proposed that “when the stomach is full, the intestines tend to be weak; if the intestines are full, the stomach is prone to be weak; the spirit is the residence when the five internal organs are stable.” Only with normal gastrointestinal function can our Qi and blood run smoothly in the human body. Thus, only when all the body organs function at a healthy level can we experience optimal cognitive ability. For people with poor gastrointestinal function, the Qi and blood cannot function normally. If the Qi in the body is disordered, night insomnia occurs. From the perspective of Western medicine, this phenomenon has been explained as the function of the brain-gut axis (Wang et al., 2014). Additional research results has revealed the importance of healthy gastrointestinal function, and even promoted the concept that the intestine was the “second brain” (Li et al., 2020). Disorders associated with the neuroendocrine network of the gastrointestinal tract can reduce sleep time by influencing the central nervous system. This relationship has been associated with Ghrelin, which is an essential brain-gut peptide. Ghrelin is expressed in both the gastrointestinal tract and the central nervous system (Cassoni et al., 2001). Ghrelin has been shown to affect the entire brain-gut axis, with sleep quality as one of its effects (Weikel et al., 2003). Researchers also reported that Ghrelin has a significant effect on insomnia and anxiety (Steiger, 2006). Based on the information noted above. Therefore, even if constipation and chronic atrophic gastritis did not present a stable risk in our study, we still proposed that these factors were significant for post-stroke care in stroke patients. In summary, keeping the gastrointestinal tract healthy and having regular bowel movements were beneficial. However, after adjustment, the differences were not significant, showing that the risk was unstable, which could be explained by the fact that these factors were affected by other variables in the specific patient situations. Thus, we suggested that researchers investigate these factors in the future and try to exclude the influence of other factors.

With respect to the current understanding of PSI risk factors, few studies have explored the relationship between cardiovascular disease and sleep, but clinicians have realized that they are correlated. In our study, the risk of coronary heart disease was not statistically significant (crude HR = 1.13, *P* = 0.146, adjusted HR = 0.94, *P* = 0.456). Therefore, we could not draw a definitive conclusion based on this study. We included coronary heart disease in our analysis because, as early as 1983, the association between coronary heart disease and sleep disorders has been observed, which was primarily related to sleep-disordered breathing. Sleep-disordered breathing results in reduced night blood oxygen saturation and reduced cardiac output causing arrhythmia, blood pressure fluctuations, and increased sympathetic nerve activity (Šiarnik et al., 2015). MRI evidence also revealed the presence of ischemic white matter changes (Robbins et al., 2005). Because few studies have investigated this factor and the mechanism of the interaction between the two is unclear, a prospective randomized controlled trial is needed to clarify this association. Thus, additional research is needed to determine whether sleep intervention could affect heart metabolism (Grandner et al., 2012).

The relationship between insomnia and stroke is highly complicated. It involves hemodynamics, nerve damage, metabolic changes, endothelial damage, and inflammatory changes (Hepburn et al., 2018). The inflammatory response in stroke is considered an important pathological mechanism leading to cerebral ischemia, which mainly influences the reduction of interleukin 1 beta (IL-1beta) gene expression and damages hippocampal neurons. Lack of sleep also enhances the gene expression of anti-inflammatory factors IL-6 and IL-10, which have been associated with improved ischemic prognosis. The unbalanced Th1/Th2 cell response derived from CD4+T cells was considered the primary cause of the immune imbalance. Acupuncture regulates the immune system by enhancing the activity of natural killer cells and regulating the balance of a subgroup of Th1/Th2 cells (Kim and Bae, 2010). Acupuncture promotes the release of related neurotransmitters in the central nervous system, acting on the sympathetic nervous or parasympathetic nervous systems to regulate the immune system (Mori et al., 2002). The use of acupuncture acupuncture reduced the loss of hippocampal neurons in rats and reversed the 2VO-induced upregulation of TXNIP, NLRP3, caspase-1, and IL-1β, which alleviated oxidative stress and decreased the circulation of inflammatory factors (Du et al., 2018). Therefore, treatment of PSI with acupuncture might directly reduce the brain damage caused by stroke or indirectly treat insomnia caused by anxiety and depression. Acupuncture eliminated the risk factors that produced sleep disorders and regulated the balance of the autonomic nervous system. We found that patients who received acupuncture after stroke exhibited more stable moods, a more objective state of mind, and a more positive attitude. These factors were closely related to sleep and rehabilitation after stroke. Thus, these factors were clear advantages derived from using acupuncture to treat stroke.

Because our research was of a clinical retrospective nature, in the process of collecting patient information, we found that the acupuncture prescriptions of each patient were not the same However, the main core acupoint composition was similar. The acupoints frequently used clinically include Baihui (GV 20), Sishencong (EX-HN 1), Shenting (GV 24), Neiguan (PC 6), Shenmen (HT 7), Shenmai (BL 62), and Zhaohai (KI 6). The importance of these acupoints also has been reported by other researchers, and their treatment mechanisms have been explored (Gao et al., 2013). Baihui and Shenting belong to the Du meridian. Chinese medicine believes that the Du meridian directly enters the brain and is closely related to the brain and mental activity. The “Compendium of Materia Medica” calls “the brain the home of the mind.” Therefore, acupuncture at acupoints on the Du meridian could effectively treat mental disorders such as insomnia. Although Sishencong, Shenting, and Shenmen belong to different meridian points, they are similar in that they are related to the spirit, which implies that they are closely related to the spiritual activity of the human body.

The clinical experience of Chinese medicine indicates that these acupoints have excellent therapeutic effects. Usually, after completing the entire acupuncture protocol, the needle is left in the patient for 30 minutes to achieve a better treatment effect. This is related to the function of the body's defensive Qi, which is the right Qi to protect the human body, as it enhances the body's immunity (Gao et al., 2010). According to the “Huangdi Neijing,” it takes approximately 30 minutes for Wei Qi to circulate once through the body. Clinical experience has revealed that when the frequency of treatment is carried out three or four times a week for 30 minutes each time, the maximum beneficial treatment effect is achieved. This is the most commonly used treatment frequency in Chinese medicine clinics. Because the patient's acupuncture points involve the head and upper and lower limbs, the supine position is most frequently used to facilitate the acupuncture protocol in the clinic.

It should be noted that stroke is a dynamic process. The relationship between insomnia and stroke is not limited to the relationship just between just these two conditions. Other post-stroke complications can aggravate the harm caused by stroke by affecting insomnia, such as post-stroke fatigue, depression after stroke, and others. These factors are related to mood disorders, neurological sequelae, neuropsychological sequelae, and some overlap with post-stroke insomnia (Winward et al., 2009). Also, insomnia was affected by the interaction of external factors, whose influence might be more complicated and challenging to identify and control. For example, some PSI was caused by the patient's environment or exposure to related drugs (Autret et al., 2001). Fortunately, acupuncture has been shown to provide a considerable therapeutic advantage in the treatment of such diseases. For stroke groups in general, priority should be given a primary complaint of sleep disorder (Cavalcanti et al., 2012). With early detection of PSI is often treatable, and early treatment could avoid re-injury to the patient. This is consistent with our concept of preventive treatment. Thus, early effective treatment could improve the well-being and quality of life of stroke patients.

## Limitations

Our research presented several shortcomings. First, our study was a single-center study that took place at the First Affiliated Hospital of Guangzhou University of Chinese Medicine. This hospital primarily utilizes Chinese medicine treatment methods. To better strengthen the controls in such a study, multi-center research should be carried out in the future. Second, our research results suggested that acupuncture exhibited a preventive treatment advantage in treating PSI. However, no specific acupoints were provided. To guide the clinical application of acupuncture more accurately, additional acupuncture details related to the PSI treatment should be presented. Third, we did not classify the severity of the PSI. Future research is needed to provide a more precise classification of this factor. Thus, a deeper understanding of the connection between acupuncture and insomnia could be elucidated and provide more focused guidance. Finally, as all clinical retrospective studies could be improved, it is necessary to increase the sample size to provide stronger, evidence-based conclusions.

## Data Availability Statement

The original contributions presented in the study are included in the article/supplementary material, further inquiries can be directed to the corresponding author/s.

## Ethics Statement

The studies involving human participants were reviewed and approved by The First Affiliated Hospital of Guangzhou University of Chinese Medicine. Written informed consent for participation was not required for this study in accordance with the national legislation and the institutional requirements.

## Author Contributions

XQ conceived the initial research direction and drafted the manuscript. XQ, NH, JY, FY, YL, and XZ was responsible for data collection and inputted and sorted during the whole process. XQ and NH was responsible for verifying and checking the correctness of the data. XQ and NH was responsible for data statistics. XZ was responsible for the guidance and suggestions of the entire research process. All the above authors have contributed to this research and completely agree for publication.

## Conflict of Interest

The authors declare that the research was conducted in the absence of any commercial or financial relationships that could be construed as a potential conflict of interest.

## Publisher's Note

All claims expressed in this article are solely those of the authors and do not necessarily represent those of their affiliated organizations, or those of the publisher, the editors and the reviewers. Any product that may be evaluated in this article, or claim that may be made by its manufacturer, is not guaranteed or endorsed by the publisher.
